# Involving Motor Capabilities in the Formation of Sensory Space Representations

**DOI:** 10.1371/journal.pone.0010377

**Published:** 2010-04-28

**Authors:** Daniel Weiller, Robert Märtin, Sven Dähne, Andreas K. Engel, Peter König

**Affiliations:** 1 Department of Neurobiopsychology, Institute of Cognitive Science, University of Osnabrück, Osnabrück, Germany; 2 Department of Neurophysiology and Pathophysiology, University Medical Center Hamburg-Eppendorf, Hamburg, Germany; University of California Irvine, United States of America

## Abstract

A goal of sensory coding is to capture features of sensory input that are behaviorally relevant. Therefore, a generic principle of sensory coding should take into account the motor capabilities of an agent. Up to now, unsupervised learning of sensory representations with respect to generic coding principles has been limited to passively received sensory input. Here we propose an algorithm that reorganizes an agent's representation of sensory space by maximizing the predictability of sensory state transitions given a motor action. We applied the algorithm to the sensory spaces of a number of simple, simulated agents with different motor parameters, moving in two-dimensional mazes. We find that the optimization algorithm generates compact, isotropic representations of space, comparable to hippocampal place fields. As expected, the size and spatial distribution of these place fields-like representations adapt to the motor parameters of the agent as well as to its environment. The representations prove to be well suited as a basis for path planning and navigation. They not only possess a high degree of state-transition predictability, but also are temporally stable. We conclude that the coding principle of predictability is a promising candidate for understanding place field formation as the result of sensorimotor reorganization.

## Introduction

In order to predict neuronal response properties on the basis of their sensory input, a number of generic sensory coding principles have been proposed. The most prominent are sparse coding [1] and temporal coherence [2], [3]. The former is closely related to efficient coding and redundancy reduction [4], the latter to slow feature analysis and stability [5], [6]. These principles successfully capture some characteristic neuronal properties of early sensory cortices [7], [8], [9]. Employing them in a hierarchical structure leads to high-order sensory representations such as place fields [2], [10]. Overall, the description of neuronal response properties by quantitatively defined principles has proven to be quite successful in furthering our understanding of sensory processing [11].

The principles of sparseness and temporal coherence were originally formulated without explicit reference to the behavioral repertoire of the agent. Agent behavior influenced learning in the sensory hierarchy only indirectly through the statistics of the sensory input selected by action. Specifically, active sensing and passive replay of recorded sensory events would have the same effect on learning. It is far from obvious whether variation in the statistics of the passively received sensory stimuli of different mammals is large enough to explain the striking cross-species differences in the layout of sensory processing [12].

Common to these principles of sensory coding is that good sensory representations should emphasize *relevant* aspects of the sensory information. Given identical sensory input, different aspects might be relevant for different agents. Hence, relevance is beyond the scope of passively perceived sensory input. Rather, relevance is grounded in behavior of an agent. The algorithm proposed herein aims to find sensory representations that are relevant given the behavioral capabilities of a situated agent, interacting with its environment. In accordance with König and Krüger [13], we posit that these sensory representations should maximize the predictability of sensory state transitions given a motor action.

The proposed optimization algorithm is based on a general, graph-theoretic framework and, in principle, is able to deal with arbitrary sensors and effectors. The algorithm divides the agent's sensory space into discrete states. The agent learns the transition probability between these states by executing action primitives from its motor repertoire in an exploratory fashion. These state transition probabilities are then evaluated with respect to their predictability and the degree of their decorrelation. Here, predictability refers to the sparseness of the distribution of potential target states. A probability distribution is sparse when it contains many low values (ideally zeros) and relatively few high values (ideally a single entry of unit value). The closer the state transition dynamics of a sensorimotor system are to this ideal, the more deterministic it becomes and the more predictable it is. The predictability of a state is defined by the sparseness of transition probabilities, averaged over actions.

The degree of decorrelation of two states quantifies the dissimilarity of their transition probability distributions. In order to increase predictability and decorrelation, the algorithm iteratively modifies the current sensory space discretization by either cutting or merging states. This optimization process is guided by a number of rule-based heuristics and increases an objective function based on the measures of predictability and decorrelation.

We applied this optimization algorithm to an agent moving in a virtual two-dimensional environment. Here, the agent's sensory space was spanned by its position within the environment. We found that the proposed algorithm successfully improved the predictability and decorrelation of the agent's sensory space representation. Optimized states corresponded to spatially compact, isotropic regions of the agent's two-dimensional maze environment, much like discrete place fields [14]. These properties of optimized states were robust and only slightly affected by the choice of motor parameters. In contrast, the size of optimized states was strongly dependent on motor parameters, while the spatial distribution of states depended on motor parameters as well as on environment type. We also demonstrate that representations optimized for predictability are a suitable basis for path planning and navigation, offering good navigability while keeping the representation low-dimensional. Furthermore, we test our claim that including knowledge of the agent's motor repertoire in the optimization process improves the quality of the resulting representations. We compare optimized-state configurations generated from purely sensory data to those generated from sensorimotor data. We find that the latter possess significantly higher predictability values and are spatially more compact and smaller. Including knowledge of motor capabilities also reduces the variability of the outcome of the optimization process. A comparison between states resulting from optimizing temporal stability [10] and those resulting from optimizing predictability, revealed that both representations were nearly equally temporally stable, whereas predictability was markedly higher in the latter.

## Methods

Here we introduce the optimization algorithm for the reorganization of a simulated agent's sensory space representation. The goal of this algorithm is to arrive at a representation that renders the state transitions caused by a motor action as predictable as possible, while representing transition dynamics in a decorrelated, i.e. efficient, manner.

The algorithm reaches this goal by dividing sensory space into disjoint discrete states. We will refer to these states as macrostates and to a specific sensory space discretization as a macrostate configuration. The dynamics of a sensory state transition resulting from an action are captured in the form of macrostate-transition probabilities. Thus, agent behavior is modeled as a Markov decision process. The predictability and the degree of decorrelation of a macrostate configuration are determined by examining the transition probabilities between its constituent macrostates.

### Simulating agent behavior

**Figure 1:**
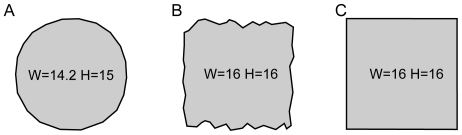
The shapes of the arenas used in the simulations. Widths and heights of the bounding boxes are given in step-length units (see Method section).

We simulated an agent moving in a number of two-dimensional environments. It could execute any one of eight action primitives corresponding to traveling a fixed distance (parameterized by *step-length*) in one of eight equally spaced directions in the interval from 0° to 360°. Step-length and movement direction were subjected to additive Gaussian noise (parameters: *step-length noise σ*_SL_, *angular noise σ*_A_). The agent's sensory space was spanned by its position in the two-dimensional environment. The action parameters were varied to explore their influence on the outcome of the optimization process. The statistics presented in the result section are based on five optimization runs for every combination of step-length (1, 2, 3 and 4), *σ*_SL_ (1/15, 2/15 and 3/15) and *σ*_A_ (2°, 6° and 12.3°). As the agent's behavior was simulated, spatial units were chosen such that the smallest step-length was set to one, and thus these units have to be seen in relation to the size of the environment. We used three maze arenas of comparable size (with bounding boxes of ca. 20×20 step units): one circular, one square with straight walls and one roughly square with irregular walls (see figure 1). In summary, the simulated agent can sample sensory events (in our case, position in continuous space) by executing action primitives from a finite set of action primitives that are subject to noise.

### Macrostate-transition probabilities

We assumed that the agent possessed the means to acquire knowledge of macrostate-transition probabilities [15]. It could do so by simply running a random exploratory motor program if these probabilities required updating as a result of macrostate modification. Because it is computationally not efficient to re-simulate this exploration procedure after each iteration of the optimization algorithm, we made use of a shortcut by introducing *microstates*. Please note that this shortcut exists for reasons of algorithmic efficiency alone and that it can be replaced by any means to sample macrostate transition probabilities.

Microstates are the atomic elements of a very fine grained, regular discretization of sensory space. We define the size of a microstate to be 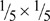. The transition probabilities in sensory space were thoroughly sampled once by a simulated agent executing actions primitives in random sequence. The agent did not avoid wall contact but reflected off the wall in such a manner that the reflection angle was equal to the incident angle. This random exploratory motor program was applied for each environment and for each set of motor parameters. Even though the simulated agent moved in continuous space, the changes in position caused by its actions were recorded in a discretized form in the microstate transition table which, when normalized to unit sum, would yield *microstate transition probabilities*. The exploration was concluded when the agent had visited 95% of the states at least 500 times. In the experiments presented here, the agent executed on average 624 (±41) actions on each microstate. Because macrostates can be seen as disjoint sets of microstates, transition probabilities at the macrostate level can be computed from microstate transition probabilities.

The transition probabilities between microstates or macrostates following an action are stored in the corresponding transition matrices. The macrostate-transition matrix is referred to as *TM*, with each entry *TM_i,j,k_* representing the transition probability from macrostate *i* to macrostate *j* using action *k*. Similarly we define the transition matrix of the microstates *tm*. Each entry of this transition matrix *tm_i,j,k_* defines the probability to make a transition from microstate *i* to microstate *j* with action *k*.

We will now demonstrate how to compute the transition probabilities from a particular macrostate (source state) i to all other macrostates j for a particular action k. To do so, we interpret the microstate-transition probabilities for the action under consideration as a directed, weighted graph. The microstates that are contained within the source-macrostate *i* now are “injected” with an “activity”, i.e. probability mass, of unit sum (see equation 1).
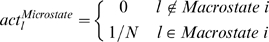
(1)Here, *N* denotes the number of microstates in macrostate *i*. This initial activity is propagated along the microstate-transition graph once for each action *k*, resulting in a new microstate-occupancy probability distribution (see equation 2).
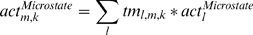
(2)Here, the probability mass flows from the microstates associated with the source to all other microstates in a manner proportional to the probability of reaching the microstates if action *k* were executed. For each potential goal macrostate *j*, the summed activity of its member microstates corresponds to the transition probability from the source macrostate to that macrostate (see equation 3).
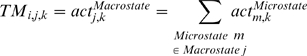
(3)After this computation has been performed, *act_j,_*_***k***_ ***^Macrostate^*** contains the probability to transit from macrostate *i* to macrostate *j* by performing action *k*. In order to fill the complete macrostate-transition matrix, this procedure has to be repeated for each action and each macrostate.

### Optimization

Following the computation of macrostate-transition probabilities, these were evaluated with respect to their predictability and the degree of their decorrelation. This enabled the optimization algorithm to modify the macrostate configuration in such a way as to improve these measures. We define the predictability *pred_i_* of a macrostate *i* as the sparseness of its transition probability distribution to other macrostates j, averaged across actions k. As proposed in [16], the sparseness of such a discrete distribution was measured by its Euclidean norm (see equation 4). Please note that for each k and i, TM_i,j,k_ is a probability distribution over target states j and therefore 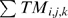 is unity. In contrast, its Euclidean norm, i.e. pred_i_, lies in the interval [0,1].
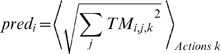
(4)


In order to calculate the degree of decorrelation of a macrostate *i*, *decorr_i_*, one first has to compute the decorrelation value between the transition probabilities of all macrostates *i* and *j*. The decorrelation of two macrostates was specified to be the inverse of the not centered correlation coefficient of the corresponding pair of transition probability distributions, averaged across actions *k* (equation 5). Here we used the not centered correlation coefficient to measure the similarity between transition probabilities. We chose this measure as the uncentered correlation coefficient between two orthogonal probability distribution vectors is zero, whereas the normal (mean-free) correlation coefficient would be negative, which is undesirable given the design of the objective function (equation 7).
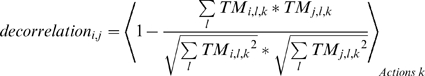
(5)This results in a square matrix with each entry representing the average decorrelation of macrostate *i* and macrostate *j*. We define the degree of decorrelation of macrostate *i*, *decorr_i_*, to be the minimum decorrelation of that macrostate with any other macrostate *j* (see equation 6).
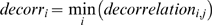
(6)A maximally predictable, yet trivial macrostate configuration would consist of a single state with the probability of remaining in this state being 1. In order to prevent the algorithm from arriving at this trivial solution, self-connection strength (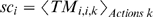) is to be kept low for all states. To obtain an overall measure of macrostate quality, we introduced the objective function, Ψ. The Ψ value of an individual macrostate *i* is a weighted sum of its predictability and minimum decorrelation (*decorr_i_*) scaled by the inverse of its self-connectivity strength. After an optimization run has been concluded, the macrostate configuration with the highest average Ψ is chosen to be that run's result.
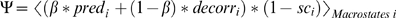
(7)


Here, β takes values between 0 and 1. For the present study, we set β to 0.8 (see Result section for details). Ψ ranges from 0 to 1, while the maximum of 1 assumes zero self-connectivity. This maximum is, however, never reached in practice as the presence of motor noise prevents unit predictability values.

Computing the derivative of the objective function with respect to changes in the macrostate configuration is not feasible due to the high dimensionality of configuration space. As a consequence, the optimization algorithm is not driven by the gradient of Ψ, but by a number of rule-based heuristics: If a macrostate has low predictability, it is split in two. If a pair of macrostates is highly correlated, they are merged. The two antagonistic operations, “Merge” and “cut” can be understood as an iterative local coarsening or refinement of the sensory space discretization aimed at producing state configurations with high average Ψ.

The merge operation combines all microstates of two macrostate into one macrostate, the cut operation clusters the microstate population contained in a macrostate into two populations, yielding two new macrostates.

A successful cut should result in an increase of the predictability of the current macrostate setup by ensuring that both of the new macrostates possess sparser transition probabilities than their predecessor. An effective way to do so is to find a cut that maximizes similarity of microstate-to-macrostate transition probability, i.e. projection similarity (equation 8), within clusters and minimizes this similarity between clusters. The projection similarity *conn_i,l_* between a pair of microstates *i* and *l* is computed by correlating the microstate-to-macrostate transition probability vectors for the two microstates (equation 8).
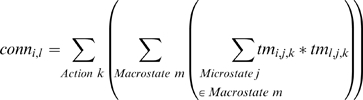
(8)Based on this similarity measure, we applied the *normalized cut* graph-partitioning algorithm [17] to split the microstate population into two clusters with maximal within-cluster projection similarity and minimal across-cluster projection similarity.

In each iteration the optimization algorithm has to decide whether to cut or to merge as well as which macrostates to subject to the selected treatment. A number of simple rule-based heuristics were employed to make these decisions. The first thing following macrostate evaluation was to compile lists of possible *merge-candidates* and *cut-candidates*. The list of merge-candidates initially contained all macrostate pairs. During each iteration a macrostate can only be modified once. Consequently, the list was refined by resolving intersections between macrostate pairs by removing the macrostate pair with the higher decorrelation value. Thus, highly correlated pairs of macrostates had a higher merge priority than less correlated pairs. Also candidate pairs were discarded that, if merged, would create a macrostate whose self-connection strength was its largest transition probability. The cut-candidate list contained all macrostates characterized by a predictability smaller than the median of predictability values of the current macrostates configuration. Once the candidate lists were composed, the algorithm chose to carry out the merge operation if the mean decorrelation was lower than the mean predictability. If mean predictability was lower than mean decorrelation, the cut operation was executed instead.

In addition to these rules, we introduced an upper boundary for the possible number of macrostates to prevent the algorithm from reaching the trivial macrostate configuration where each macrostate consists of a single microstate. This boundary was periodically changed every 20 iterations from 1000 macrostates to 500 macrostates to 10 macrostates to prevent the algorithm from oscillating. We tested a number of different boundary values but did not observe any systematic influence on the number of macrostates in the optimized configurations. Each optimization run was initialized using a randomized macrostate configuration. After some thousand iterations (on average 3000 iteration steps), the optimization process was concluded.

In summary, each iteration of the optimization process consists of 3 steps. First, the macrostate transition probabilities of the current configuration of macrostate are calculated by using the microstate transition probabilities. Second, based on the macrostate transition probabilities the macrostate configuration is evaluated according to predictability and decorrelation. Third, with respect to the decorrelation and predictability values, the current macrostate configuration is modified.

### Analyzing spatial structure of macrostates

In the present case, sensory space is equivalent to the two-dimensional environment the agent behaves in. Therefore, each macrostate occupies a distinct spatial region within that environment. To investigate the spatial structure of these regions, we applied three measure of region analysis [18]. The *area* of a macrostate is defined by the number of microstates in it. *Solidity* is a measure of macrostate compactness and computed by dividing the area of that macrostate by the area of its convex hull. The *eccentricity* of a region is defined by the ratio of the distance between the foci of a fitted ellipse and the length of its major axis. *Roundness* is an inverse measure of macrostate eccentricity (*roundness = (1 – eccentricity)*). Further, in some of the spatial analysis of the macrostate, we weighted solidity and roundness by the logarithm of macrostate area in order to prevent macrostates consisting of single microstates from receiving high scores (see Result section).

To directly compare the topographical distribution of two macrostate configurations, we applied a measure of spatial similarity. First, we constructed a binary spatial-occupancy map for each macrostate. Then, each occupancy map from the first macrostate configuration was compared to each map of the second configuration by computing the not centered correlation coefficient. To get from this one-to-many comparison to an injective one-to-one comparison, each macrostate in the smaller configuration was associated with the macrostate in the other configuration which was spatially most correlated to it. Consequently, there remained a single correlation value for each macrostate in the smaller configuration. These values were averaged to yield the final measure of configuration similarity.

### Planning & Navigation

We investigated whether the state space representations generated by our algorithm are useful to an agent interacting with its environment by testing how well these representations can serve as a basis for path planning and navigation. As a comparison, we also assessed the navigability of control-state configurations. In this section we describe the generation of the control-state configurations as well as navigation and path planning.

**Figure 2:**
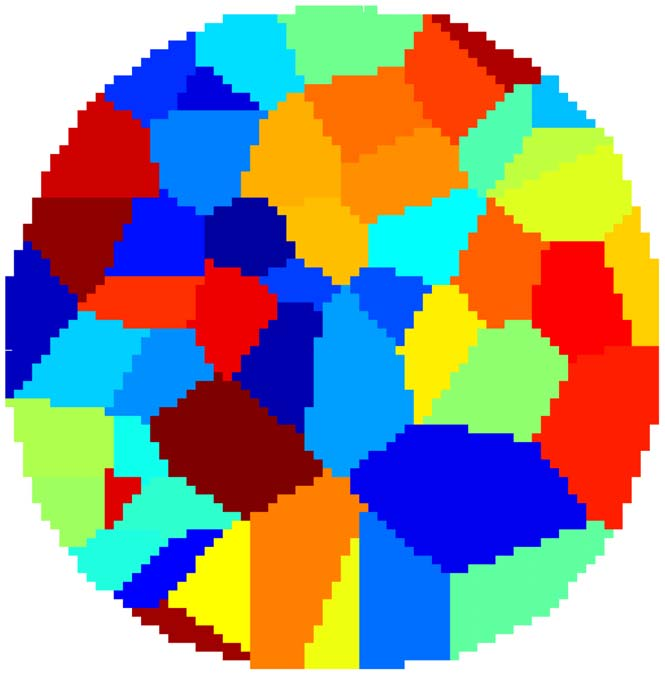
Exemplary control macrostate configuration. A control macrostate configuration generated by randomly placing 50 Gaussian curves within the environment and applying a winner-take-all operation. Each color represents a different macrostate. Although the small differences in the hues in the display might be interpreted otherwise, all macrostates are simply connected.

To create a control-macrostate configuration consisting of a given number of (N) macrostates, one randomly places N two-dimensional Gaussians within the environment and applies a winner-take-all function to arrive at a discrete partitioning of the environment into N regions. The state configurations thus generated are qualitatively similar to those produced by our optimization algorithm (see Result section): they are solid and approximately convex (see figure 2). The essential difference between control configurations and optimized configurations is the precise placement of these convex, compact states.

The goal of the navigation tasks is to reach a region of connected microstates the same size as the average macrostate of the optimized state configuration. In a planning stage, the agent uses its knowledge of macrostate-transition probabilities and a form of Dijkstra's algorithm [19] to compute a state-action function, mapping each macrostate to the action that is most likely to lead to the goal. To get from its starting position to the goal region, the agent employs a closed-loop control scheme: After the execution of every action, it consults its policy to select the best action for the currently occupied macrostate.

After each action, a macrostate-occupancy probability distribution is computed along with the probability that the agent has reached the goal region (*goal-occupancy probability*). These probabilities are calculated by propagating state-occupancy probability mass along the graph according to the state-action function. First, unit activity is injected at the start microstate. Second, the activation is propagated to the connected microstates and multiplied by the transition probabilities associated with the action dictated by the state-action function. This yields an updated state-occupancy-probability distribution. Propagation is repeated until the summed activity in the goal region, i.e. the goal-occupancy probability, reaches a threshold of 0.95. The navigability of a macrostate configuration was quantified by the number of steps it took to reach this threshold.

Additionally, we computed the entropy of the macrostate-occupancy distributions of the agent as it moved towards the goal region. Here, entropy is a measure of path dispersion or localizability: low entropy values indicate that the agent follows a stereotypical and reproducible sequence of states. To compare an optimized configuration to a control configuration, we computed the ratio of the corresponding state-occupancy entropy values averaged across the number of actions needed on average to reach the criterion was 18. We used only agents with the smallest step-length motor parameter, such that a reasonable number of steps would be required to reach a goal.

## Results

We applied the optimization algorithm to the sensory spaces of a number of agents with varying motor parameters, and analyzed the algorithm's behavior as well as the representations generated by it. Optimized macrostate configurations were analyzed with respect to their topographical properties, tested for their usefulness as a basis of path planning and navigation, and compared to states generated by a temporal stability objective.

### Analysis of the objective function

**Figure 3:**
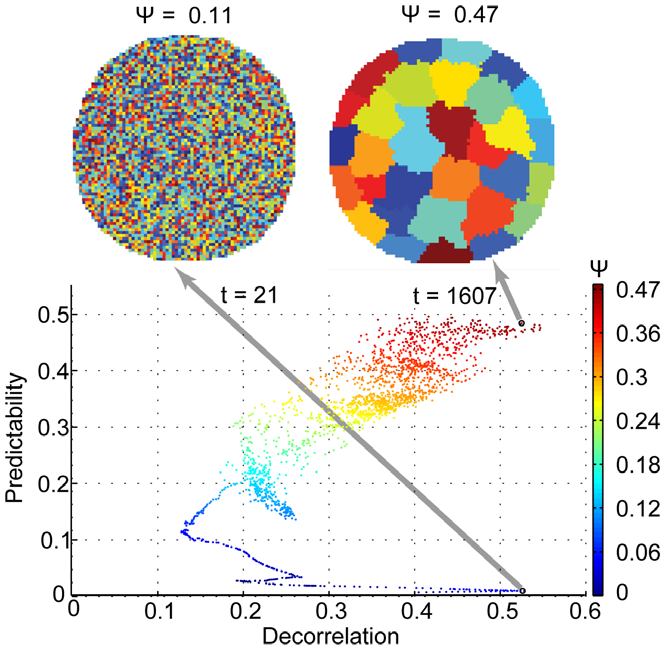
The relation between the objective function Ψ, predictability and decorrelation for one exemplary optimization process. Each point represents the average of these measures for one iteration step of the optimization run. The two insets show the spatial layout of the macrostate configurations at selected iterations where each macrostate identity is coded by a color.

Does the optimization algorithm successfully increase the objective function? To answer this question, we examined how the objective function Ψ and its components, decorrelation and predictability, evolve during the course of optimization. As the optimization process does not directly optimize Ψ using gradient-based techniques, the optimal-macrostate configuration (highest average Ψ) does not need to occur at the end of the optimization process. Usually, Ψ increased for the first 100 iterations and then began to change discontinuously. The optimization time-stamps in figure 3 illustrate this for an exemplary optimization run. Across all optimization runs, the random initial macrostate configuration at the beginning of the run had a mean predictability of 0.01 (standard deviation ± 0.005) and mean decorrelation of 0.45 (standard deviation ± 0.14). The value of Ψ averaged over all initial conditions was 0.12 (standard deviation ± 0.03). Across all optimized macrostate configurations, the predictability value was 0.516 (standard deviation ± 0.069), the decorrelation value was 0.48 (standard deviation ± 0.08) and Ψ was 0.43 (standard deviation ± 0.06). Note that, given the difference between initial (random) and optimal-macrostate configuration, the dynamic range of decorrelation is larger than it appears to be. Because a random macrostate configuration could be rather decorrelated, there were iterations during which the decorrelation was lower than the initial value. Note also that self-connection strength stayed very small (below 0.05) throughout the optimization process. It becomes clear that the optimization process successfully increased the objective function Ψ as well as its major components, most notably predictability.

**Figure 4:**
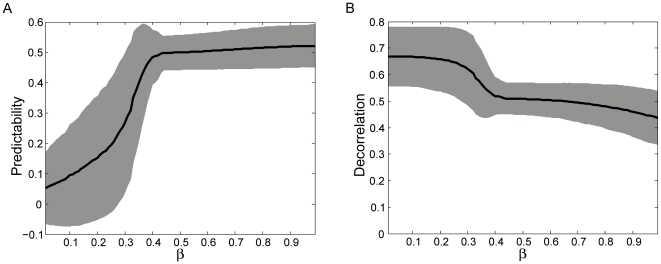
Influence of the weighting factor β (equation [1]) on the predictability and decorrelation values of the optimized macrostate. The black line represents the mean predictability (A) and decorrelation values (B) over all optimization runs for the corresponding β values. The grey regions represent the area within ± one standard deviation around the mean values.

We examined how strongly the results of the optimization algorithm depend on the relative weighting of predictability and decorrelation (see equation 1). To this end, we varied the weighting factor β from 0.01 to 0.99. It should be kept in mind that β does not affect the optimization process, but only the assessment of the quality of the optimized state configuration. The mean decorrelation and predictability values of the optimal-macrostate configurations over all optimization runs are shown in figure 4. For most values of β, including the default value used in the present investigation of β = 0.8, the resulting macrostate configurations have similarly high predictability values. We found that for β<0.33 (standard deviation ± 0.08), a qualitative change of the optimized state configuration took place. Predictability values dropped sharply while decorrelation increased (see figure 4). In this domain, the optimization process returned degenerate macrostate configurations that were very similar to the random initial macrostate configuration and all optima were found within the first 58 iterations. This is due to the disproportionate weighting of decorrelation over predictability and to the fact that randomly initialized macrostate configurations possess highly decorrelated transition probabilities (see figure 3). Thus, macrostate configurations at the beginning of the optimization process receive very high objective function values. An analysis of the compactness values of the optimal-macrostate configurations yielded consistently high compactness values (similar to the values presented in the next subsection) for β values larger than 0.33, and consistently smaller values below this threshold. Thus, above a β value of 0.33, the algorithm produces spatially compact macrostate configurations with a high degree of predictability.

**Figure 5:**
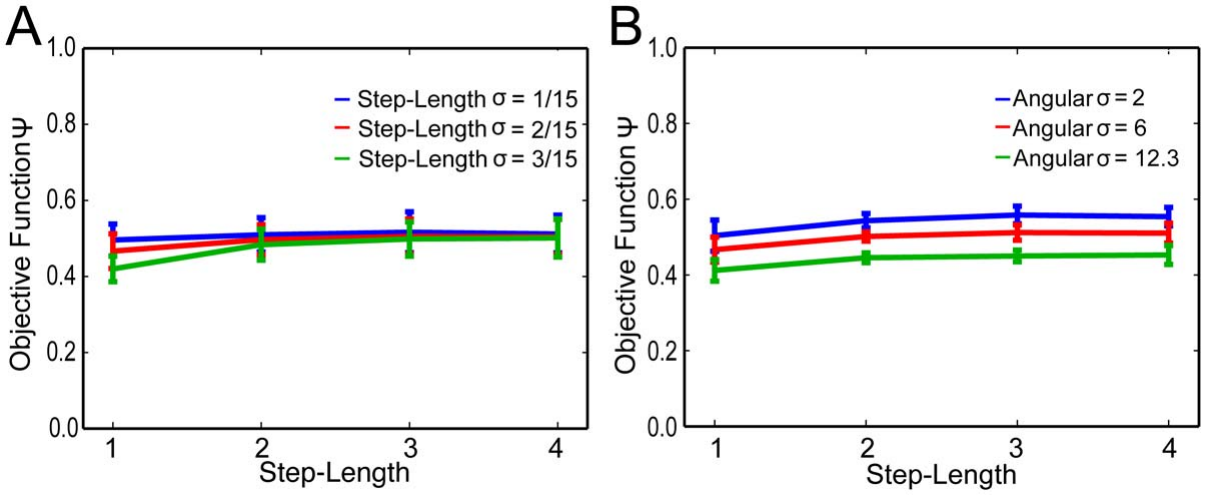
The influence of motor parameters on the average Ψ of optimized macrostate configurations.

As a next step, we investigated whether the objective function is influenced by parametric properties of the behavioral repertoire. Step-length and step-length noise have a small influence on the value of the objective function reached (see figures 5A and 5B). A small interaction of step-length noise and step-length can be observed at the small steps with high noise with a marginal decrease of Ψ. Furthermore, increasing the angular noise slightly decreased the objective function value at the optimized state configurations (see figure 5B). All of these noise effects were largely determined by predictability. Decorrelation was not consistently affected by noise in the motor parameters. This is quite intuitive, as the noise level strongly influences movement accuracy, ultimately limiting the degree of predictability that can be achieved. However, the effect size was small compared to the dynamic range of Ψ (see e.g. figure 3), and thus negligible. We conclude that the optimization process reliably generates state configurations with high Ψ values, and that the optimization process is robust with respect to the choice of motor parameters.

### Analysis of the spatial structure of macrostates

Each macrostate occupies a two-dimensional region of the maze environment. Here, we analyzed how properties of these regions change in the course of optimization. For the initial state configuration, the average macrostate roundness (1-eccentricity) and solidity were 0.38 (standard deviation ± 0.02) and 0.012 (standard deviation ± 0.006), respectively. The corresponding values for optimized macrostate configurations were 0.66 (standard deviation ± 0.08) and 0.89 (standard deviation ± 0.004).

**Figure 6:**
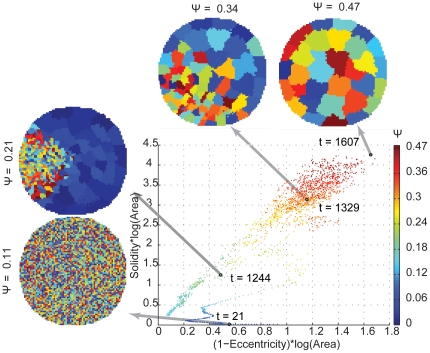
The relation between three macrostate properties: the objective function Ψ, size-weighted solidity and size-weighted roundness (1-eccentricity). Each point represents the average of these measures for one iteration step in an exemplary optimization run. The four insets show the spatial layout of the macrostate configurations at selected iterations. Macrostate identity is coded by color.

An exemplary optimization run illustrates this trend in figure 6. Each data point corresponds to a single iteration. Color codes for the averaged Ψ value over all macrostates of each macrostate configuration was associated with that iteration, while position represents size-weighted roundness and size-weighted solidity. High values of the objective function are associated with large, round and compact states. Accordingly solidity and roundness increased during the optimization process. Note that an increase in macrostate size is equivalent to a reduction of macrostate number, as the size of the environment remains constant.

**Figure 7:**
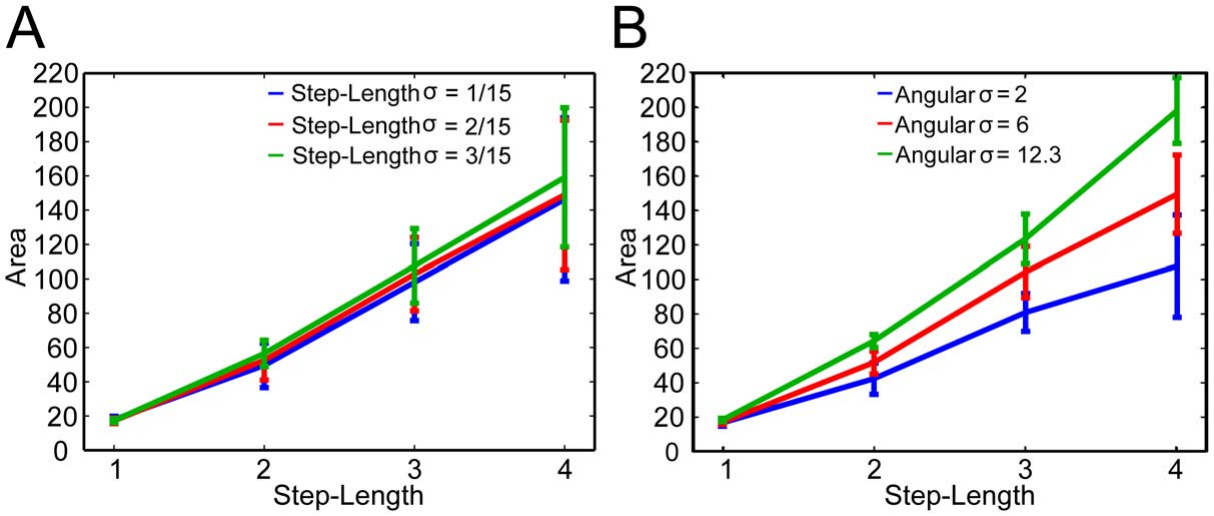
The influence of motor parameters on the average macrostate area of the optimized configurations.

Next, we investigated how macrostate spatial properties depend on the choice of motor parameters. Figures 7A and 7B show that the area of macrostates increases in response to an increase in step-length. In figure 7B, we see that this effect interacts with angular noise σ_A_: a larger σ_A_ leads to larger macrostates. This interaction effect is to be expected from geometrical considerations. No such effect can be observed for step-length noise σ_SL_. Neither roundness nor solidity of optimized macrostates was affected by the choice of motor parameters. There were no conclusive effects of maze type on spatial properties.

**Figure 8:**
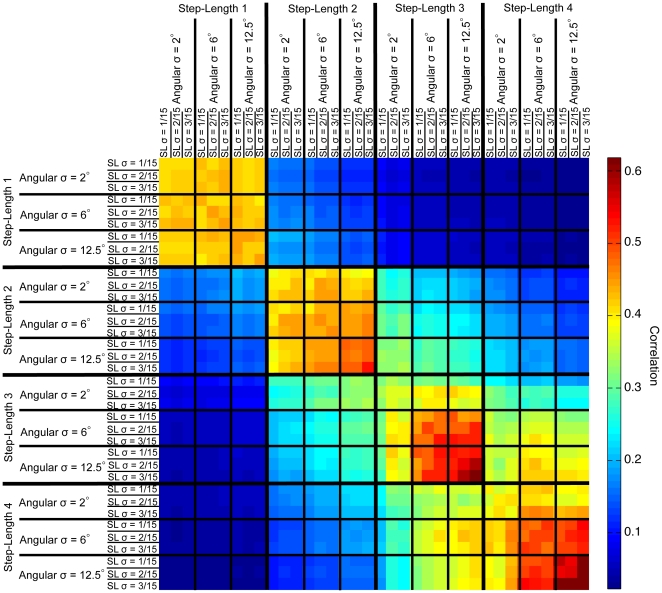
Correlation between the optimized macrostate configurations. The correlation matrix showing the topographical similarity between optimized macrostate configurations with different underlying action parameters. This matrix is based on the circular maze type, but is similar to those for the other mazes.

Further, we directly compared the topographical arrangement of optimized macrostate configurations for different action parameters (see Method section). We calculated the correlation coefficients between macrostate configurations with different motor parameters, as described in the Method section. Those coefficients can be arranged in groups of high values with similar step-lengths and low values with different step-lengths, as shown in figure 8. A comparable, yet weaker, tendency was observed for angular noise, such that groups of coefficients with the same angular noise have higher values than groups with different angular noise. Step-length noise, on the other hand, was not a consistent predictor of topographical similarity. Comparing different types of environment while keeping motor parameters constant revealed that the macrostate distributions of the square environment and the irregular square environment were moderately correlated (0.34±0.06, mean ± standard deviation). The correlation of macrostate topography in the circular environment was less correlated with the other two (0.12±0.02, mean ± standard deviation, circular vs. irregular; 0.11±0.02, mean ± standard deviation, circular vs. square).

We conclude that optimized macrostate configurations possess large, spatially compact macrostate distributions. Macrostate size is inversely related to motor precision: large step-lengths in combination with large angular noise terms lead to large macrostates. Furthermore, similar environments lead to similar spatial distributions of macrostates. We hypothesized that it is this adaptation of macrostate size and distribution to motor parameters and environment type that renders the objective function robust with respect to these issues. This dependence is intuitive, as coarsening the sensory space discretization maintains predictability in the face of increased motor noise.

### Usefulness for Planning & Navigation

Are macrostate configurations that are generated by our predictability-optimization algorithm useful to an agent that is actively engaging with its environment? We investigated whether these representations form a suitable basis for path planning and navigation, and compared their performance to that of control maps generated by randomly distributing compact macrostates (see Method section).

For every optimized macrostate configuration, we conducted 100 navigation trials. In each trial, a goal region had to be reached from a randomly chosen position outside it. Navigation performance was evaluated on the optimized map and on a control map containing the same number of macrostates. Consequently, every trial yielded a pair of state-occupancy probability distributions that evolve over time. From these distributions, the probability that the agent has arrived in the goal region (goal-occupancy probability), as well as the entropy of the spatial probability distribution of the agent, was computed.

#### Navigation performance

To investigate the navigational performance, we counted the number of actions required to reach the goal with a probability of at least 0.95. The mean number of steps required to reach the 0.95 criterion was 16.43 (standard error of mean 0.09) for optimized maps, and 18.00 (standard error of mean 0.12) for control maps. The difference between the number of steps in the control and optimized macrostate configuration was 1.6 (standard error of mean 0.13; sign-test significant at 0.05 level). Thus, compared to the control configuration, optimizing predictability of the macrostate configuration significantly improved navigational performance.

To assess the reproducibility of the state-sequence leading to the goal, we computed the ratio of the state-occupancy entropies of optimized and control configurations (see Method section). Because ratios tend to produce outliers, robust statistical measures were used here. Across all trials, the median of this ratio was 0.93 with an inter-quartile range of 0.41. Thus the entropy of the optimized state configuration is higher than in the control case. We conclude that during navigation, optimized configurations allow for more reproducible state-action sequences than control configurations do.

#### Relation between navigation performance and the number of macrostates

Does an increase of the number of macrostates in the control configuration compared to the optimized configuration result in better navigational performance? To investigate this question, we varied the number of macrostates within the control configuration from 50% to 150% of the number of macrostates in the optimized configuration. We investigated ten equally spaced points within this interval. Again, we counted the number of steps required to reach a goal-occupancy probability of 0.95. The navigational performance changed as a function of the number of macrostates with a slope of 6.16 per unit (units given by the ratio of the number of macrostates in the control configuration to the number in the optimized configuration). The dependence was well approximated by a linear fit (r = 0.99). Thus, at 50% fewer macrostates, an average of 5.62 more steps are needed to reach the goal, while with 50% more macrostates, an average of 0.43 fewer steps are needed. In summary, increasing the number of macrostates results in an increase in navigational performance. On average, control configurations containing less than 117% of the macrostates in the optimized configuration offer worse navigability than the optimized configuration. Beyond 139%, performance of the control configuration may exceed that of optimized configurations. Thus, in order to increase the navigational performance based on a control configuration, we have to increase the number of states.

In practice, increasing the number of macrostates comes at a high cost. As the number of macrostates increases, to approximate the transition probabilities between macrostates by means of an exploratory motor program (see Method section) becomes an increasingly daunting task. Therefore, representational parsimony is important. Furthermore, when the motor system is inherently noisy, improving state-space resolution will not improve navigation performance beyond a certain level. Thus, the choice of the number of macrostates must be balanced between representational parsimony and navigability. In contrast, in the optimized configuration, the number of macrostates is given by the motor parameters of the agent; it is not a free parameter. Further, the optimization of predictability yields a higher navigability compared to the control configuration of the same dimensionality. This suggests that the optimized macrostate configurations are well balanced between navigability and representational parsimony.

In summary, although these macrostates are optimized with respect to an agent's motor capabilities and not to a particular navigational goal, optimized configurations offer better navigability than control configurations. Additionally, in the optimized state configuration, the agent traverses a more reproducible sequence of states on its way to the goal. We conclude that optimized state configurations are well balanced between navigability and representational parsimony, and thus are suitable for path planning and navigation.

### The importance of knowing the agent's actions

Here, we investigate whether representations generated with knowledge of the action repertoire are different from those generated without it.

We collapsed the three-dimensional microstate-transition matrix *tm* (see Method section) along the action dimension. As a consequence, the distinction between motor actions was lost, while knowledge about sensory state transitions remained. These purely sensory transition probabilities where then used as input to the optimization algorithm. This is done for all step-lengths and noise parameters, but only for the circular arena (N = 180). We then compared these configurations to those generated with knowledge of motor actions on the basis of their average predictability, decorrelation, self-connection strength, roundness and compactness.

Sensory-only transition probabilities contain the pooled transition probabilities associated with each action. Thus, sensory-only states are, by virtue of their construction, connected to more states than states associated with individual actions. Consequently, the sparseness, i.e. predictability (equation 4), of sensory-only state configurations is lower. To assure a fair comparison, we evaluated the optimized sensory-only configurations as if they were based on the full microstate-transition matrix *tm*. More precisely, we first applied the optimization algorithm to the collapsed (sensory-only) transition matrix. The resulting macrostate configuration map together with the uncollapsed (differentiating between actions) microstate transition matrix was then used to compute macrostate transition probabilities for every action. The objective function values of these transition probabilities were then computed to objective function, predictability and decorrelation values of the state configurations generated with uncollapsed transition probability matrices. In summary, the optimized sensory-only configurations where *generated* with a transition matrix collapsed along the action dimension, but *evaluated* using the full matrix.

**Figure 9:**
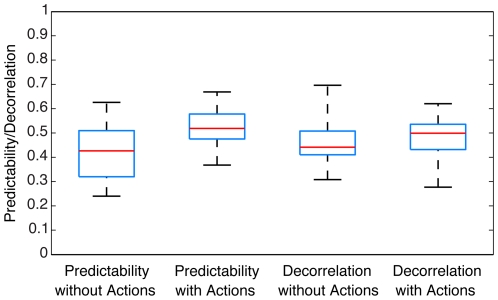
Comparison of the predictability and decorrelation of state configurations generated with and without the knowledge of the agent's motor capabilities. The horizontal lines in each box represent the 25^th^, 50^th^ (median) and 75^th^ percentiles, while the black whisker bars mark the range of the data.

As can be seen in figure 9, state configurations generated from sensorimotor data achieved higher predictability and decorrelation values than configurations generated from purely sensory data. The average difference of predictability between these two populations was a highly significant 0.10 (0.08 standard deviation, Wilcoxon sign-rank test p<0.05). In order to quantify the difference between the populations, we evaluated the classification performance of the predictability values between them by analyzing the area under ROC. For the predictability values, the area under ROC was 0.75. Furthermore, the average difference between the decorrelation values was 0.05, and highly significant (Wilcoxon sign-rank test p<0.05), although a minor classification performance compared to the predictability values was obtained (area under ROC = 0.59).

We also found that configurations based on sensorimotor data were spatially more compact (mean difference 0.35±0.11, area under ROC = 1). In addition, dividing the average macrostate area of configurations generated with motor information, by the average macrostate area of sensory-only configurations, revealed that the latter were consistently larger. This ratio increased as a function of step-length (step-length 1: 3.06, step-length 2: 3.51, step-length 3: 4.51, step-length 4: 4.47). Not only is the average macrostate in a sensory-only configuration larger than the average sensorimotor macrostate, but its size is also much more variable. Again, the ratio of the standard deviations of the area distributions is dependent on the step-length, yet for all step-lengths we obtained a larger standard deviation in the sensory-only condition (step-length 1: 53.56, step-length 2: 29.62, step-length 3: 23.61, step-length 4: 19.87).

We conclude that including knowledge of an agent's motor capabilities in the optimization process has an influence on the resulting representations. These representations possess higher predictability and decorrelation values than sensory-only representations. Their constituent macrostates are smaller and more compact. Finally, configurations based on sensorimotor data exhibit much less variability in the average macrostate size, indicating that the optimization process becomes more reliable when motor information is present.

### Comparison of temporal stability and predictability

Wyss et al. [10] found that place fields form an optimally temporally stable neural representation of the video stream recorded by a robot moving in a two-dimensional environment. Since both [20] and [13] point out the connection between temporal stability and predictability, we compared the place fields reported in [10] to our macrostates in terms of their temporal stability and state-transition predictability.

Before making this comparison, we had to address some technical differences. To convert continuous place field activity distributions to a discrete partitioning of space, we applied a winner-take-all process. The result was of the same formal type as the macrostate distributions. Hence, once motor parameters had been selected, Ψ could be computed. For the opposite transformation, i.e. to map discrete macrostates to a continuous activity distribution, we fitted a Gaussian to the largest connected component of each macrostate, adapted its falloff behavior to that of the activity distributions reported in [10] and iteratively refined the distribution locally until a winner-take-all operation yielded the original discrete macrostate configuration. These two algorithms allowed an unbiased comparison of continuous place field activity distributions and discrete partitioning of space.

Once we had mapped a macrostate configuration to a place field configuration, we could assess its global stability by averaging the stability objective with respect to each cell's activity [10]. We evaluated the temporal stability of place-cell activity as the robot moved through the environment using a motion generator similar to that used in [10]. The movement consisted of a random sequence of translation and rotation combined with obstacle avoidance. The probability of switching between translation and rotation was set to 0.1. The stability was calculated by the activity values of the place cells at the position of the agent within the environment. Here, stability is a measure between zero and infinity, where zero would denote perfect stability. The reported values were normalized such that a random state configuration, similar to the initial condition of the optimization algorithm described above, receives unit stability.

For the actual comparison, we chose a place field distribution reported in [10] and a macrostate configuration of a matching square maze. The latter was obtained by applying our optimization algorithm to the sensory space of an agent whose motor parameters were extrapolated from figure 7. We computed ten optimized macrostate configurations using these parameters. The optimization process yielded configurations consisting of an average of 23.5±5.1 (mean ± standard deviation) macrostates. This is close to the 25 place fields reported in [10].

Here we compared the predictability and stability values of the macrostate configurations to the place fields reported in [10]. The scale of the predictability values ranged from 0 (not predictable) to 1 (highly predictable), while the stability values varied between 0 (perfect stability) and 1 (stability of a randomly distributed macrostate). The difference between the mean stability values of the macrostate configurations and the stability values of the place fields of [10] was very small, 0.0130±0.0013 (mean ± standard deviation), while the stability values of the macrostate configuration were slightly higher. To control for possible biases induced by the mapping from discrete macrostates to continuous distributions, we discretized the reported place fields using a winner-take-all operation, and then turned them into continuous place fields, using the same algorithm that was used to turn macrostates into place fields. With this technique, the difference between the mean stability values of the macrostates configurations, resulting from optimizing predictability, and the place fields of [10] was 0.0126±0.0013 (mean ± standard deviation). Thus the macrostate configurations were slightly more stable than the one optimized for stability. The average difference between the predictability of the macrostate configurations resulting from optimizing predictability and the place fields of [10] was 0.3680±0.003 (mean ± standard deviation). The macrostate configurations had higher predictability values compared to those of [10]. In comparison, the difference in predictability values between the place fields of [10] and a random distribution of macrostates was 0.172±0.001 (mean ± standard deviation), with higher predictability values for the place fields of [10]. Thus both optimization processes result in an increase in predictability and stability values compared to a random distribution, while optimizing with respect to predictability also leads to an increase in temporal stability, whereas the inverse is not the case.

## Discussion

The proposed optimization algorithm reliably generates sensory space representations with predictable and decorrelated state-transition probabilities. The macrostates that constitute these representations are spatially compact and comparable to place fields. The algorithm is robust with respect to a variety of parameters of the agent's motor apparatus and of the objective function. Yet, the size and topographical distribution of macrostates is adapted to the agent's motor capabilities as well as to its environment. We showed that representations optimized for predictability can be used as a basis for path planning and navigation, and offer greater navigability and representational parsimony than control configurations of compact, solid macrostates. We found that it is important to incorporate knowledge about the agent's motor capabilities into the optimization process. The absence of such motor information leads to state configurations of lower average predictability and decorrelation, whose constituent macrostates are larger, less compact and more variable. In addition, the variability of the average macrostate size is increased when motor information is not present. By comparing macrostates to the temporally stable place fields reported in [10], we found that states optimized with respect to predictability are also temporally stable.

A number of issues concerning the optimization process have to be addressed. First, the algorithm is guided by rule-based heuristics informed by predictability and decorrelation; it does not truly maximize the Ψ value. It may not converge to a stable fixed point, but maintain oscillations of finite size. Indeed, increasing the number of iterations beyond a certain limit did not yield higher Ψ values. Furthermore, up to now, we did not compute the upper boundary of possible Ψ values for a certain set of motor parameters and a certain environment. Approximating this boundary would provide a useful termination criterion. Even without termination criteria, different optimization runs result in similar optimized macrostate configurations for the same motor parameters, and thus represent a reproducible final-state configuration. Second, the algorithm assumes that the transition probabilities between macrostates are known for each action. Here, the simulated agent learned these probabilities by executing an exhaustive exploratory motor program. It is not yet clear how the optimization algorithm might perform if the transition probabilities were only poorly explored or whether it is applicable in an online learning scenario. The third issue is the assumed generality of our algorithm. Even though the sensory space of our simulated agent appears to be simplistic, a mapping between this sensory space and that used in [10] can be achieved by taking an appropriate set of unique camera views and mapping them to the corresponding spatial positions (see e.g. [21]). As the algorithm only takes into account the transition probabilities between sensory states, it does not matter from which sensory organ these states originate. Hence we consider the independence of a specific topological embedding to be the most outstanding and important feature of the presented algorithm.

State-aggregation techniques used in reinforcement learning are similar to our idea of creating a state-space representation that enables an agent to effectively interact with its environment. During reinforcement learning, an agent learns from reinforcement signals that pertain to the consequences of its interaction with the environment. The goal of this learning is to derive a control policy for the agents' state-space, which allows it to maximize expected reward, as in the solution of the Bellman equation [22]. Clearly, choosing a suitable state-space representation is just as important as choosing the learning algorithm operating on it. A number of state-aggregation techniques attempt to solve the former problem by formulating the search for a suitable state space as an optimization problem. In a theoretical study, Singh and colleagues [23] suggest that the best state-space representation is the one for which the solution to the Bellman equation can best be approximated. In practice, this theoretically sound idea is often re-framed to simply state that an optimal state space should increase the expected reward [24], [25], [26]. Reynolds [27] proposed a state-space representation based on clustering, i.e. merging, of states according to the action-value function resulting from, e.g. Q-Learning. Chrisman [28] developed a state-aggregation technique quite similar to our own approach. He argues that transitions between states resulting from an action should be as predictable as possible. If the transition dynamics are unpredictable for a particular state, this state should be split in a manner as to render each of the new states more predictable. This is basically the description of the “cut” procedure in our algorithm. Chrisman, however, does not introduce a merge operation, and this reduces the number of possible state configurations compared to our algorithm, which modifies the states by a cut-and-merge procedure. In contrast to these state-aggregation techniques, our algorithm organizes the sensory space with respect to the agent's motor capability in a completely unsupervised fashion, independent of a certain task or reward structure.

Recent modeling studies suggest that place cells can be regarded as temporally stable representations of the video stream recorded by a behaving agent [9], [10]. In these studies, the behavior of the agent only indirectly influences place field formation, as it influences which sensory inputs are sampled from the environment. In contrast, our algorithm directly involves motor action in the creation of sensory representations. We found that properties of the motor apparatus are a key determinant of the size and number of states generated by our algorithm. In contrast, the number of place fields reported in [10] is predetermined by the number of output neurons. Thus, the number of place field–like representations produced by our algorithm emerges from the statistical structure of sensorimotor space and is not a free parameter. There is evidence for the biological reality of integration of motor signals and sensory input in the formation of place fields. Terrazas and colleagues [29] investigated the influence of self-motion information on the formation of place fields in behaving rats. They investigated the difference between place fields formed by rats that were able to actively explore their environment and place fields formed by rats that were passively transported through the same environment. Even though visual and vestibular input were similar in both conditions, the place fields formed by actively moving animals contain more information about that animal's position within the environment than place fields formed on the basis of passively received sensory input. This shows that motor signals are involved in the formation of sensory representations found in biology.

Earlier studies proposed an involvement of motor signals in place field formation via path-integration processes. Path-integration refers to the continuous integration of self-motion cues into the representation of one's orientation and position with respect to a certain starting point. Both sensory input and motor output are transformed into such self-motion cues before being processed by the integration mechanism. In combination with landmark-based mechanisms, path-integration has been proposed as a basis for place field formation [14]. In contrast, we maintain that motor signals directly, i.e. without being converted to self-motion cues, influence the formation of sensory representations (such as place fields) to increase the predictability of the sensory outcome associated with a particular motor action.

In this study we find that there is a relationship between the amount of noise present in action execution and the coarseness of the sensory representation: more noise leads to larger states (see, e.g. figure 7). From this finding, we can make the prediction that a similar relation might hold between the precision of the motor system of an animal and the spatial extend of its place-fields. Using a virtual reality apparatus for rats [30], it is possible to manipulate the degree of noise in the mapping from motor action to sensory input. As it is also possible to record place-cell activity from a rat immersed in the virtual environment, our prediction can be tested quite easily given the right equipment. An analogous prediction could be made for the influence of step-length, i.e. movement speed, on the average size of place-fields.

The presented algorithm is a first approach to utilize predictability as a generic coding principle. As predicted in [13] and [20], we found that optimizing for predictability leads to representations that are temporally stable as well. This suggests that predictability is a more powerful coding principle than temporal stability. Consequently, unsupervised learning algorithms maximizing predictability of sensorimotor state transitions are promising candidates for general models of neural coding.
